# Media-fasting in children and adolescents: a prospective study of screen-free day intentions across age groups

**DOI:** 10.1007/s00431-025-06633-4

**Published:** 2025-11-25

**Authors:** Silke Schwarz, Arndt Büssing, Benjamin Streit, David Martin

**Affiliations:** 1 Institute for Integrative Medicine, Chair of Medical Theory, Integrative and Anthroposophic Medicine, Department of Human Medicine, Faculty of Health, University of Witten/Herdecke, Herdecke, Germany; 2 Professorship Quality of Life, Spirituality and Coping, University of Witten/Herdecke, Herdecke, Germany; 3 University Children’s Hospital Tuebingen, University of Tuebingen, Tuebingen, Germany

**Keywords:** Digital screen media, Screen-free days, Intentions, Children, Parents, Survey data

## Abstract

Intensive use of screens during childhood is associated with health risks. This brief report evaluates whether a media-fasting intervention increases the number of screen-free days and intentions to reduce screen media use in different pediatric age groups. A 6-week voluntary intervention, including pre- and post-intervention surveys, was conducted in six pediatric practices (North Rhine-Westphalia, spring 2019). Among 407 children and adolescents initially enrolled, 229 completed the post-intervention survey (43.7% drop-out); 169 were matched for pre/post analysis. “Screen-free days” were defined as 24 h with no recreational screen exposure. Data were self-reported; SES was not assessed. Adjusted mixed-effects models were employed to appropriately account for the longitudinal structure of the data across measurement occasions. Younger children reported significantly more screen-free days than adolescents, both pre- and post-intervention. Intentions for screen-free days increased after the intervention, especially in the 6–9 and 10–13 year groups. Most participants already avoided screens during meals and at night. Neither age nor gender was significantly associated with screen time.

*Conclusion*: Media-fasting may promote more frequent screen-free days, particularly in younger children. Owing to substantial attrition, the absence of SES data, and reliance on self-reports, findings should be interpreted with caution.

**Table:** 

**What is Known:** • *Excessive childhood screen use is linked to adverse health outcomes.* • *Interventions for reducing screen time have shown inconsistent efficacy.*
**What is New:** • *This study demonstrates age-dependent effects of a brief media-fasting intervention.* • *Simple family-targeted approaches may promote screen-free days in younger youth.*

**Supplementary Information:**

The online version contains supplementary material available at 10.1007/s00431-025-06633-4.

## Introduction

Excessive screen media use in children and adolescents represents a global public health challenge, being associated with adverse effects on physical, psychological, and social well-being [1, 2]. Leading organizations, including the American Academy of Pediatrics (AAP) and German scientific societies, recommend limiting or even avoiding screen-based electronic media for young children, especially those under the age of two, and urge strict daily limits and high-quality content for older children [3–5]. Despite these guidelines, screen time has continually increased in recent years, often exceeding international recommendations [6].

Efforts to reduce screen time via interventions have thus far yielded inconsistent and often modest effects. Practical, family-based behavioral targets that move beyond quantitative screen time, and that can be flexibly adapted to daily life, are rarely studied across pediatric age groups.

This brief report therefore aims to evaluate whether a short, family-focused “media-fasting” intervention can increase the number of screen-free days, and the intention to reduce screen media use, among children and adolescents of various age cohorts.

## Methods

A 6-week voluntary media-fasting intervention was conducted in six pediatric practices in North Rhine-Westphalia, Germany, during spring 2019. Families with children and adolescents were recruited for active participation through pediatricians’ offices. No formal exclusion criteria were applied. Written informed consent was obtained from legal guardians for both pre- and post-intervention data collection. The study protocol was approved by the ethics committee of Witten/Herdecke University (application no. 194/2018) and conducted in accordance with the Declaration of Helsinki.

Participants received a “media-fasting” kit that included recommendations, a calendar with 44 surprise doors, and two sets of questionnaires (for parents and for children, before and after the intervention). The intervention encouraged families to reduce recreational screen time by reflecting on habits and considering alternative joint activities in nature, sports, culture, and nutrition. Children under 6 years required parental assistance to complete the questionnaire due to limited reading comprehension.

The child and adolescent questionnaire included items regarding independence in filling out the instrument, five items on intended screen use during the intervention, two items on intentions to implement screen-free days (using an 8-point response scale, 0–7 days per week), and additional measures of well-being, physical activity, and outdoor behavior. “Screen-free days” were defined as 24 h without any recreational screen exposure, as explicitly communicated to all participants. Socioeconomic status (SES) was not assessed.

Statistical analysis involved descriptive statistics (frequencies for categorical, means ± SD for numerical variables). Age group comparisons used the Mann–Whitney *U* test, as many variables were not normally distributed. A significance level of *p* < 0.01 was applied to compensate for multiple testing; eta squared (*η*^2^) was used as an effect size indicator, with thresholds for small (< 0.06), moderate (0.06–0.14), and strong (> 0.14) effects. Data were analyzed using SPSS 28.0. A linear mixed-effects model was applied with screen time as the dependent variable. Fixed effects included the measurement timepoints (before vs. after intervention), age, and gender. Pair identification was included as a random intercept to control for within-pair correlation. Parameter estimation was performed using restricted maximum likelihood (REML) [7]. Due to the study design and survey nature, missing data were neither imputed nor adjusted.

## Results

A total of 407 children and adolescents were enrolled, of whom 229 (56.3%) returned the post-intervention questionnaire, resulting in a 43.7% drop-out rate. After matching pre- and post-intervention surveys, data from 169 participants (42% girls, 58% boys; mean age 9.4 ± 3.9 years) were analyzed. Distribution across age cohorts was as follows: < 6 years (18.5%), 6–9 years (37.5%), 10–13 years (27.4%), and 14–18 years (16.7%). Most questionnaires for children under 10 years were completed with parental assistance. No participants were excluded based on age, although the method section states a minimum age of 6 years; some younger children participated if parents supported questionnaire completion.

Prior to the intervention, 29% of children and adolescents intended to reduce TV time; 72.5% already avoided screens during meals, 71.2% avoided screens before breakfast, and 80.0% kept mobile phones out of their bedroom during sleep. Only 24.7% reported no screen time after dinner, although 42.9% stated an intention to avoid it. The majority (63%) had no media-free days per week at baseline.

Intentions for screen-free days before the intervention were highest in young children and lowest in adolescents (*p* < 0.001, *η*^2^ = 0.166). After the intervention, intentions remained highest among the youngest and lowest in adolescents (*p* < 0.001, *η*^2^ = 0.219). For actual media-free days per week, children under 10 reported 2–3 days before the intervention, whereas adolescents reported nearly none (*p* < 0.001, *η*^2^ = 0.208). After the intervention, children under 10 reported 3 days and adolescents reported 1 day per week (*p* < 0.001, *η*^2^ = 0.105).

Overall, matched analysis revealed that median intended media-free days increased from 2.58 to 2.70 per week and actual media-free days from 1.15 to 2.09 per week following the intervention (*p* < 0.001, *η*^2^ = 0.054). Increases were most pronounced among 6–9 and 10–13-year-olds. No significant changes were observed in physical activity or quality of life scores.

**Fig. 1:**
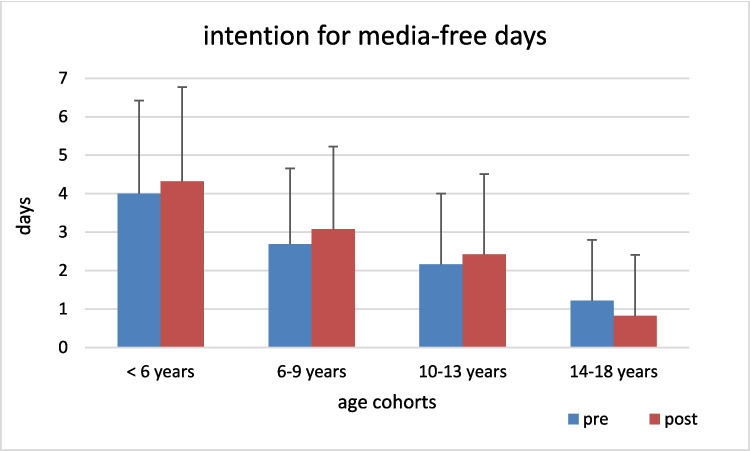
Change in mean number of media-free days per week by age group, before and after intervention (bars, mean ± SD)

Figure 1 illustrates the change in mean number of media-free days by age group before and after the intervention.

Detailed sample characteristics and full outcome tables are provided in the supplementary materials (Supplementary Tables S1–S2). Supplementary Figure S1 shows the current media-free days by age group before and after intervention.

Neither age (*β* = 0.07, *p* = 0.29) nor gender (*β* =  − 0.13, *p* = 0.75) was significantly associated with screen time. The random variability among pairs was considerable compared to residual variance, indicating notable pair-specific differences.

## Discussion

This brief report demonstrates that a short, family-focused media-fasting intervention was associated with significant increases in both the intention to have screen-free days and the actual frequency of media-free days among children and adolescents, particularly in younger age cohorts. These findings support the notion that simple, pragmatic interventions in the pediatric field can motivate families toward healthier media habits, even though absolute changes (especially in older children) remain modest [8–10].

Younger children consistently showed higher intentions and actual numbers of screen-free days, in line with developmental theory suggesting that younger children are more likely to comply with family routines and parental modeling. The age-dependent effect may also reflect differences in motivation, environmental support, and peer influences, which grow in importance during adolescence. Prior research has shown that interventions to reduce screen time among youth often lead to short-lived or limited effects, with the greatest impact observed in children whose baseline use is moderate or low. It is notable that, while intentions to reduce TV time were common, fewer participants reported intentions to reduce overall screen use after dinner—an established predictor of problematic media use.

There are several notable limitations. First, the absence of a control group, reliance on self-reported measures, and the high attrition rate (43.7% drop-out) all limit the generalizability of the findings. No adjustment for baseline screen time, SES, or potential confounders was made, and only univariate analyses were reported. Socioeconomic status (SES) may substantially influence both screen behavior and response to interventions, but was not assessed in this study. Furthermore, there was no objective measurement of actual screen time, and data on missing values were not systematically analyzed. The sample included participants younger than the stated minimum age due to practical study logistics; these cases were retained in the analysis, though they may introduce additional bias.

The findings support the efficacy of the media-fasting intervention in reducing screen time, consistent with international guidelines such as those by the American Academy of Pediatrics (AAP), the German Society for Pediatric and Adolescent Medicine (DGKJ), and recent systematic reviews on screen time interventions. These results also align with current evidence regarding the developmental benefits of reducing screen exposure for children [11–15].

Additional preventive recommendations include limiting screen time for young children to specified daily durations depending on age and encouraging media-free times to promote healthy development [11, 12].

Future research should include adjusted analyses, control groups, objective media tracking, and long-term follow-up to determine which family interventions are most effective and sustainable across developmental stages.

## Conclusion

In summary, this brief, family-centered media-fasting intervention led to a modest but significant increase in both the intention to observe screen-free days and in the actual number of media-free days among children and adolescents, primarily among younger participants. The intervention’s effects were greater for intention than for actual behavior, and substantial age-related differences were observed.

Given the high attrition, lack of socioeconomic data, absence of multivariate or longitudinal analyses, and reliance on parent- and self-reported outcomes, these results should be interpreted with caution. Nonetheless, our findings underscore the potential utility of low-threshold, scalable behavioral interventions to support healthy media habits in children and youth in accordance with international public health guidelines.

Future studies are needed to clarify mechanisms, address SES effects, and introduce objective and longitudinal measurements to better assess the efficacy and sustainability of media-reduction interventions.

## Supplementary Information

Below is the link to the electronic supplementary material.

Supplementary file 1 (DOCX 26.1 KB)

## Data Availability

No datasets were generated or analysed during the current study.
