# Supplementary material for: Media-fasting in children and adolescents: a prospective study of screen-free day intentions across age groups
Source: Eur J Pediatr. 2025 Nov 25;184(12):794. doi: 10.1007/s00431-025-06633-4 (PMC12647188; doi:10.1007/s00431-025-06633-4)
Supplement: Supplementary file 1 — (DOCX 26.1 KB) [file 431_2025_6633_MOESM1_ESM.docx]

**Supplementary Material**

**Supplementary Table S1. Description of participants before the intervention**

|  | n | % |
| --- | --- | --- |
| **Gender** |  |  |
| Female | 71 | 42.0 |
| Male | 98 | 58.0 |
| **Age cohorts** |  |  |
| <6 years | 31 | 18.5 |
| 6–9 years | 63 | 37.5 |
| 10–13 years | 46 | 27.4 |
| 14–18 years | 28 | 16.7 |
| **Filling out questionnaire** |  |  |
| With help | 114 | 67.5 |
| Without help | 55 | 32.5 |
| **Intention to reduce TV watching** |  |  |
| Not doing it anyway | 19 | 12.0 |
| For media fasting | 50 | 31.6 |
| No | 89 | 56.3 |
| **No screen time while eating** |  |  |
| Not doing it anyway | 125 | 73.5 |
| For media fasting | 36 | 21.2 |
| No | 8 | 4.7 |
| **No screen time before breakfast** |  |  |
| Not doing it anyway | 121 | 72.0 |
| For media fasting | 36 | 21.4 |
| No | 11 | 6.5 |
| **No screen time after dinner** |  |  |
| Not doing it anyway | 42 | 25.0 |
| For media fasting | 73 | 43.5 |
| No | 53 | 31.5 |
| **No mobile phone in sleeping room** |  |  |
| Not doing it anyway | 136 | 80.4 |
| For media fasting | 23 | 13.6 |
| No | 10 | 5.9 |

**Supplementary Table S2. Media-free days, quality of life, and activities before and after intervention**

| Group / Variable | Time point | Media-free days (intention, mean ± SD) | Media-free days (actual, mean ± SD) | Sporting activities (mean ± SD) | Outside time (mean ± SD) | Happy/good mood (mean ± SD) | Calm/content (mean ± SD) | Well at home (mean ± SD) | Significant change | Eta² |
| --- | --- | --- | --- | --- | --- | --- | --- | --- | --- | --- |
| All children/adolescents | Pre | 2.58 ± 2.16 | 1.15 ± 1.86 | 2.22 ± 0.63 | 2.46 ± 0.78 | 4.12 ± 0.71 | 3.88 ± 0.83 | 4.58 ± 0.64 | n.s. | .001/.054 |
|  | Post | 2.70 ± 2.36 | 2.09 ± 2.09 | 2.24 ± 0.65 | 2.62 ± 0.68 | 4.20 ± 0.73 | 4.02 ± 0.69 | 4.53 ± 0.65 | <.001 (actual days) | .054 |
| Children <6 years | Pre | 4.00 ± 2.41 | 2.52 ± 2.63 | 2.03 ± 0.66 | 2.87 ± 0.34 | 4.38 ± 0.62 | 3.93 ± 0.88 | 4.62 ± 0.56 | n.s. | .005/.019 |
|  | Post | 4.32 ± 2.45 | 3.21 ± 2.44 | 2.10 ± 0.77 | 2.97 ± 0.19 | 4.36 ± 0.62 | 4.00 ± 0.72 | 4.54 ± 0.58 | n.s. | .019 |
| Children 6-9 years | Pre | 2.69 ± 1.97 | 1.33 ± 1.69 | 2.32 ± 0.65 | 2.81 ± 0.40 | 4.05 ± 0.73 | 3.79 ± 0.93 | 4.53 ± 0.74 | n.s. | .009/.087 |
|  | Post | 3.08 ± 2.14 | 2.45 ± 1.96 | 2.31 ± 0.65 | 2.90 ± 0.30 | 4.15 ± 0.81 | 3.98 ± 0.81 | 4.54 ± 0.62 | <.001 (actual days) | .087 |
| Children 10-13 years | Pre | 2.16 ± 1.84 | 0.44 ± 0.97 | 2.26 ± 0.68 | 2.35 ± 0.60 | 4.07 ± 0.69 | 3.93 ± 0.69 | 4.64 ± 0.57 | n.s. | .004/.171 |
|  | Post | 2.42 ± 2.09 | 1.76 ± 1.86 | 2.27 ± 0.68 | 2.52 ± 0.62 | 4.21 ± 0.71 | 4.08 ± 0.58 | 4.50 ± 0.68 | <.001 (actual days) | .171 |
| Adolescents 14-18 years | Pre | 1.22 ± 1.58 | 0.11 ± 0.32 | 2.14 ± 0.45 | 1.27 ± 0.92 | 4.04 ± 0.74 | 3.93 ± 0.77 | 4.54 ± 0.64 | n.s. | .016/.087 |
|  | Post | 0.82 ± 1.59 | 0.74 ± 1.43 | 2.22 ± 0.51 | 1.85 ± 0.86 | 4.07 ± 0.66 | 3.93 ± 0.60 | 4.46 ± 0.74 | .038 (actual days) | .087 |

*Note: Eta² < 0.06 = small effect, 0.06–0.14 = moderate effect, >0.14 = strong effect. Only significant actual changes (p < .05) are bolded.*

**Supplementary Figure S1. Current media-free days by age group before and after intervention**
